# Current Safety of Renal Allograft Biopsy With Indication in Adult Recipients: An Observational Study

**DOI:** 10.1097/MD.0000000000002816

**Published:** 2016-02-12

**Authors:** Shang-Feng Tsai, Cheng-Hsu Chen, Kuo-Hsiung Shu, Chi-Hung Cheng, Tung-Min Yu, Ya-Wen Chuang, Shih-Ting Huang, Jun-Li Tsai, Ming-Ju Wu

**Affiliations:** From the National Yang Ming University (S-FT); Department of Medical Research and Center for Qualify Management, Taichung Veterans Genearl Hospital (Cheng-Hsu Chen); Division of Nephrology, Department of Internal Medicine, Taichung Veterans General Hospital (S-FT, Cheng-Hsu Chen, K-HS, Chi-Hung Cheng, T-MY, Y-WC, S-TH, M-JW); School of Medicine, China Medical University (S-FT, Cheng-Hsu Chen, M-JW); Department of Life Science, Tunghai University (S-FT, Cheng-Hsu Chen); School of Medicine, Chung Shan Medical University (S-FT, Cheng-Hsu Chen, M-JW); Rong Hsing Research Center for Translational Medicine, Institute of Biomedical Science, College of Life Science, National Chung Hsing University (M-JW); and Department of Family Medicine, Cheng Ching General Hospital (J-LT), Taichung, Taiwan.

## Abstract

Renal biopsy remains the golden standard diagnosis of renal function deterioration. The safety in native kidney biopsy is well defined. However, it is a different story in allograft kidney biopsy. We conduct this retrospective study to clarify the safety of allograft kidney biopsy with indication.

All variables were grouped by the year of biopsy and they were compared by Mann–Whitney *U* test (for continuous variables) or Chi-square test (for categorical variables). We collected possible factors associated with complications, including age, gender, body weight, renal function, cause of uremia, status of coagulation, hepatitis, size of needle, and immunosuppressants.

We recruited all renal transplant recipients undergoing allograft biopsy between January of 2009 and December of 2014. This is the largest database for allograft kidney biopsy with indication. Of all the 269 biopsies, there was no difference in occurrence among the total 14 complications (5.2%) over these 6 years. There were only 3 cases of hematomas (1.11%), 6 gross hematuria (2.23%), 1 hydronephrosis (0.37%), and 2 hemoglobin decline (0.74%). The outcome of this cohort is the best compared to all other studies, and it is even better than the allograft protocol kidney biopsy. Among all possible factors, patients with pathological report containing “medullary tissue only” were susceptible to complications (*P* < 0.001, 1.8 of relative risk).

In modern era, this study demonstrates the safety of allograft kidney biopsy with indication. Identifying the renal capsule before biopsy to avoid puncture into medulla is the most important element to prevent complications.

## INTRODUCTION

Renal biopsy remains the gold standard for diagnosis of renal function deterioration, including native and allograft kidney. In addition to diagnosis, pathological data can provide nephrologists with useful information regarding disease severity, activity, and chronicity, which can be used to guide treatment. Renal biopsy has been widely used in clinical practice for more than 100 years.^1^ This procedure is considered to be safe with acceptable complications. Percutaneous biopsy was introduced in the 1940s,^2^ and ultrasound-guided biopsy has been used since the 1950s.^3^ During the late 1980s, manual needles were gradually replaced by automated spring-loaded biopsy device,^4^ which made renal biopsy much safer and thus rapidly found wide acceptance by clinicians. The safety and complications in native kidney biopsy were established in a systematic review and meta-analysis,^5^ which included 36 studies conducted after 1990. However, the complications were highly dependent on techniques and protocols in different countries. Even in a study using data from a database of a single country,^6^ the Norwegian Kidney Biopsy Registry (1988–2010), a number of factors were still affected by the different protocols and techniques used. Percutaneous biopsy in native kidneys is thought to be a low-risk procedure without major complications. However, the risks and complications associated with allograft kidney biopsy are less well understood and should be clarified for the following reasons. First, organ shortage is a common problem worldwide, so there are relatively few studies which include data on allograft kidney biopsy. Second, recipients only have a solitary functioning kidney with less well preserved renal function. Third, studies on allograft kidney biopsy mostly date back 25 years^7^ and there are scant data from recent studies. Progress in biopsy safety was made with the introduction of the automated spring-loaded biopsy device and ultrasound guidance. The rate of complications varied according to different eras. In the past 10 years, no studies with large case numbers have been conducted to investigate allograft kidney biopsy. Fourth, the safety of allograft biopsy in children and adolescents was investigated by Franke et al,^8^ but adults were not included in the study. Finally, in the past 15 years, the largest study on adult allograft kidney biopsy was published in 2003,^9^ and reported on the safety of “protocol” biopsy in allograft kidney, rather than “indication” biopsy in allograft kidney. There are considered to be fewer complications associated with protocol biopsy than with indication biopsy because biopsy for allograft kidneys in normal situation (protocol biopsy) is safer than in problematic kidney (biopsy with indication). However, there is currently no evidence to prove this. Therefore, we conducted this retrospective study to clarify the safety of adult allograft kidney biopsy with indication in the modern era and to identify any risk factors.

## SUBJECTS AND METHODS

### Study Population

Kidney biopsy is encouraged for patients with unexplained proteinuria or renal function deterioration in Taichung Veterans General Hospital. We recruited all renal transplant recipients who underwent “allograft” biopsy between January of 2009 and December of 2014. All subjects in this study were 20 years of age or older. Patients were free from infectious disease, inflammatory disease, liver disease, or malignancy, and all were nonsmokers. All of the study procedures were conducted in accordance with the ethical standards of Taichung Veterans General Hospital and were approved by the institutional review committee (CE14172, Taichung Veterans General Hospital). It was approved after expedited review procedures in Taichung Veterans General Hospital.

### Data Collection

We collected the participants’ clinical parameters including gender, age (years), body weight (kg), timing of kidney biopsy (months after transplantation), cause of end-stage renal disease, and living donor or cadaveric donor. All diabetes mellitus (DM) patients were diagnosed according to the DM guidelines of the American Diabetes Association in 2013.^10^ Hypertension was defined as an average home systolic blood pressure greater than 140 mm Hg and a diastolic blood pressure greater than 90 mm Hg before medication, according to the definition for stage I/II hypertension set forth in the JNC-7 guidelines.^11^ Hepatitis B or C infections were confirmed by medical records, which were examined before transplantation in all recipients. Serum data were collected, serum creatinine (SCr) (mg/dL), estimated glomerular filtrate rate (eGFR)^12^ (mL/min per 1.73 m^2^), hemoglobin (g/dL), platelet (/μL), prothrombin time (seconds), and activated partial thromboplastin time (seconds). The index eGFR was calculated using the modification of diet in renal disease equation:^12^ eGFR (mL/min per 1.73 m^2^) = 186 ∗ SCr^−1.154^ ∗ Age^−0.203^ ∗ 0.742 (if female). PCR was defined by spot urine test ratio of protein and creatinine (mg/g). Immunosuppressants were defined if any drug was used for at least 3 months before renal biopsy. Postbiopsy urinary tract infection was defined as symptoms of frequency, urgency, or pyuria in recipients. Hematoma, hydronephrosis, and arteriovenous fistula were detected by ultrasound. Deep biopsy to the medulla was defined according to the results of the pathological report. Also, the diagnosis of allograft biopsy and number of glomeruli were also performed according to the pathological report.

### Biopsy Protocol

Allograft kidney biopsy protocol is frequently performed in Taichung Veterans General Hospital in all recipients with unexplained proteinuria or elevation of SCr. All patients were admitted for graft kidney biopsy. Before biopsy, recipients should control systolic blood pressure below 180 mm Hg. Antiplatelet or antithrombotic agents (eg, aspirin, omega-3 fatty acids, GP IIb/IIIa inhibitors, dipyridamole, and nonsteroidal antiinflammatory drugs) should be discontinued at least 7 days before biopsy, and warfarin should be discontinued at least 3 days before biopsy or the prothrombin time should be normalized. Pentoxifylline cannot be taken within 1 day before biopsy. Patients are instructed not to take the abovementioned drugs 7 days after biopsy. One day before biopsy, platelet, prothrombin time, and activated partial thromboplastin time should be normal. Biopsy is not performed in patients with SBP more than 180 mm Hg or abnormal coagulation function. It is important to note that Desmopressin 4 unit is infused 30 minutes before biopsy in all patients. All biopsies were performed by real-time ultrasound guidance by nephrologists. Before biopsy, the skin overlying the biopsy site should be free of signs of infection. The patient should also be able to follow simple directions (such as holding breath for at least 5 seconds). Under ultrasound guidance (3.5 MHz transducer with real-time visualization of the needle), a spinal needle was used to locate the capsule of the upper pole and to provide anesthesia for the biopsy needle tract. Two cores of renal tissue measuring 1 cm in length were generally recommended. We used an automated spring-loaded biopsy device (Bard Max-Core Disposable Core Biopsy Instrument) and the size of needle used varied between 16 and 18 G. Immediately after biopsy, we checked for any bleeding, hematoma, or arteriovenous fistula by ultrasound. All patients were required to rest in bed for at least 2 hours post-biopsy. Patients were instructed to maintain a supine posture in bed for 2 hours, and bed rest overnight was further recommended. The duration of the procedure was typically 23 minutes. To help detect bleeding and other complications, vital signs were closely monitored within 6 hours after biopsy. Blood pressure were controlled as well as possible (goal < 140/90 mm Hg). If any gross hematuria, back or abdominal pain, or dizziness or nausea were noted, urinalysis, hemoglobin, and serum sodium examinations were conducted. Ultrasound was also done to detect any complications. All patients received ultrasound regularly 1 day after biopsy without exception. That is to say, patients were under observation for at least 24 hours after biopsy. All patients were followed up to check for any complications for at least 1 year after biopsy. Complications (hematoma, hematuria, hydronephrosis, arteriovenous fistula, blood transfusion, hemoglobin decline, angiographic intervention to control bleeding, nephrectomy to control bleeding, and death) and any treatments were all recorded.

### Statistical Methods

Data are expressed as mean ± standard deviation for continuous variables and as frequency/percent for categorical variables. Demographic and clinical characteristics of the entire cohort were recorded according to years. The basic characteristics of recipients and complications divided by year of biopsy were compared by Mann–Whitney *U* test (for continuous variables) or Chi-square test (for categorical variables). Univariate logistic regression model was used to analyze the possible factors associated with complications after renal biopsy. A *P* value <0.05 was considered statistically significant. All statistical procedures were performed using the SPSS statistical software package, version 17.0 (Chicago, IL).

## RESULTS

All 1563 biopsies were selected, of which 269 allograft biopsies were selected for analysis. All basic parameters of this cohort are summarized in Table 1. The adult recipients’ cohort had a mean age of 50.3 years old and 49.4% were male. Renal functions were poor (4.02 ± 3.20 mg/dL of SCr, 26.04 ± 14.71 mL/min 1.732 m^2^ of GFR, and 6.96 ± 10.19 of PCR) because of “indication” biopsy. The timing of allograft biopsy was variable because of “indication” other than “protocol” biopsy. Most recipients (36.8%) received renal replacement therapy due to DM and were with well-controlled blood pressure (137.2 ± 16.4 mm Hg) with enlarged graft kidney size (112.4 ± 12.7 mm). Before biopsy, as mentioned in biopsy protocol, we made sure the normal coagulation functions (189,565.1 ± 60,285.7 /μL of platelet, 10.3 ± 0.9 s of PT, and 25.1 ± 3.3 s of aPTT). More than half (61.3%) of the biopsies were performed via 16-gauge automated spring-loaded biopsy needle.

**TABLE 1:**
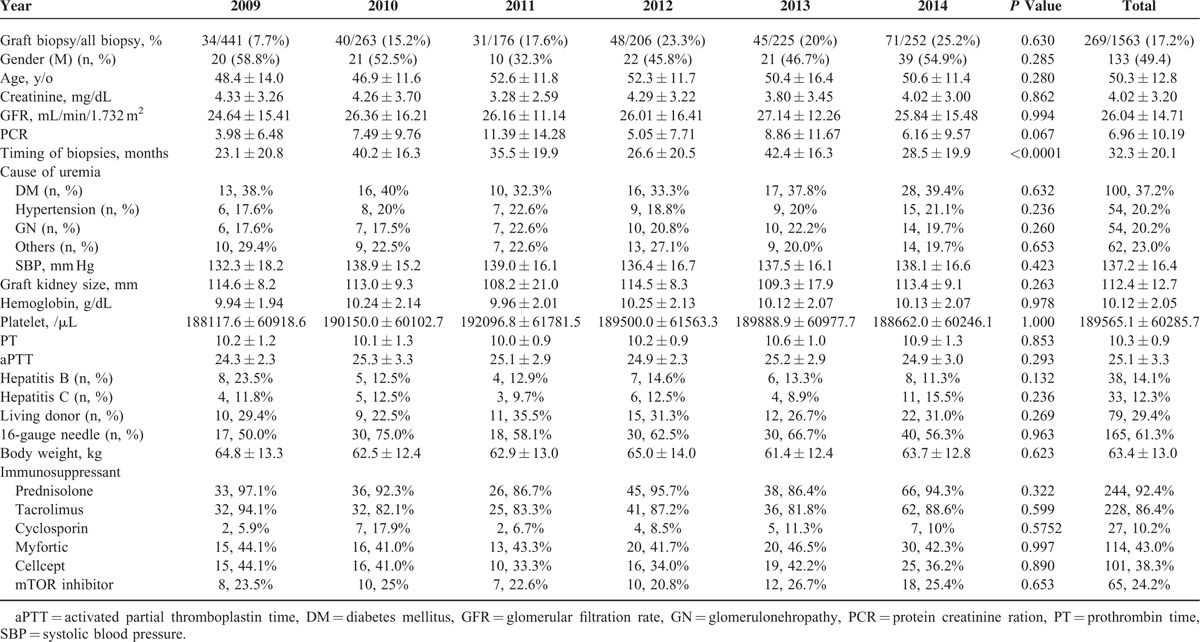
Basic Characteristics of Recipients Receiving Allograft Kidney Biopsy According to Years

Of all the 269 allograft kidney biopsies, there were 14 complications (5.2%) (Table 2). There were no statistically significant differences in all complications in the 6-year study period. The total complication rates remained unchanged during the study period (*P* = 0.236). In 2010, 2 recipients had hematomas (1 ∗ 2 cm and 1 ∗ 3 cm). Desmopressin was maintained for 2 more doses and follow-up sonography showed spontaneous resolution. No patient needed blood transfusion. Two recipients had gross hematuria and one of them resolved spontaneously. However, one of them suffered from allograft hydronephrosis. Percutaneous nephrostomy was performed 1 day after transplantation to rescue the renal function. Seven days after nephrostomy, urinary function was restored and antegrade intravenous pyelography revealed no stenosis. The catheter was removed soon afterwards. She did not receive renal transfusion in the whole course and renal function did not deteriorate due to this complication. In 2014, 1 recipient had hematoma (1 ∗ 1 cm) with spontaneous resolution after 2 more doses of Desmopressin 2 days later. Four recipients had gross hematuria and 2 of them needed 2 units of packed red blood cell transfusion because of a decline in hemoglobin (10.2 **→** 7.0 g/dL and 8.4 **→** 7.7 g/dL, respectively). No more invasive procedures were necessary. In total, complications comprised only 3 cases of hematoma (1.11%), 6 gross hematuria (2.23%), 1 hydronephrosis (0.37%), and 2 hemoglobin decline (0.74%). No patient had nausea or vomiting so there were no cases of hyponatremia. The total complication rate was 5.20%. With respect to all treatments, 1 needed percutaneous nephrostomy (0.37%), 8 needed Desmopressin (2.97%), and 2 needed a blood transfusion (0.74%). No arteriovenous fistula, graft loss, or patient death was noted and no patients needed angiographic invention or nephrectomy to stop bleeding.

**TABLE 2:**
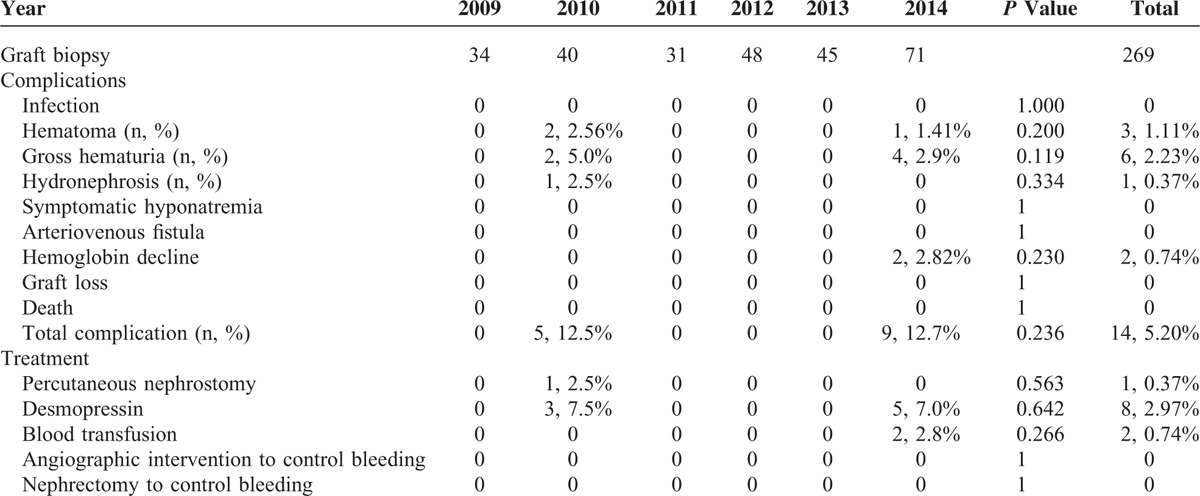
Complications and Treatments of Allograft Kidney Biopsy According to Years

Recipients with or without complications were analyzed in Table 3. Of all the potential factors, a pathological report noting “medullary tissue only” was a risk factor for postbiopsy complications (*P* < 0.001, 1.8 of relative risk) by univariate logistic regression. Other recipient conditions or biopsy-related factors did not appear to affect the risk of postbiopsy complications.

**TABLE 3:**
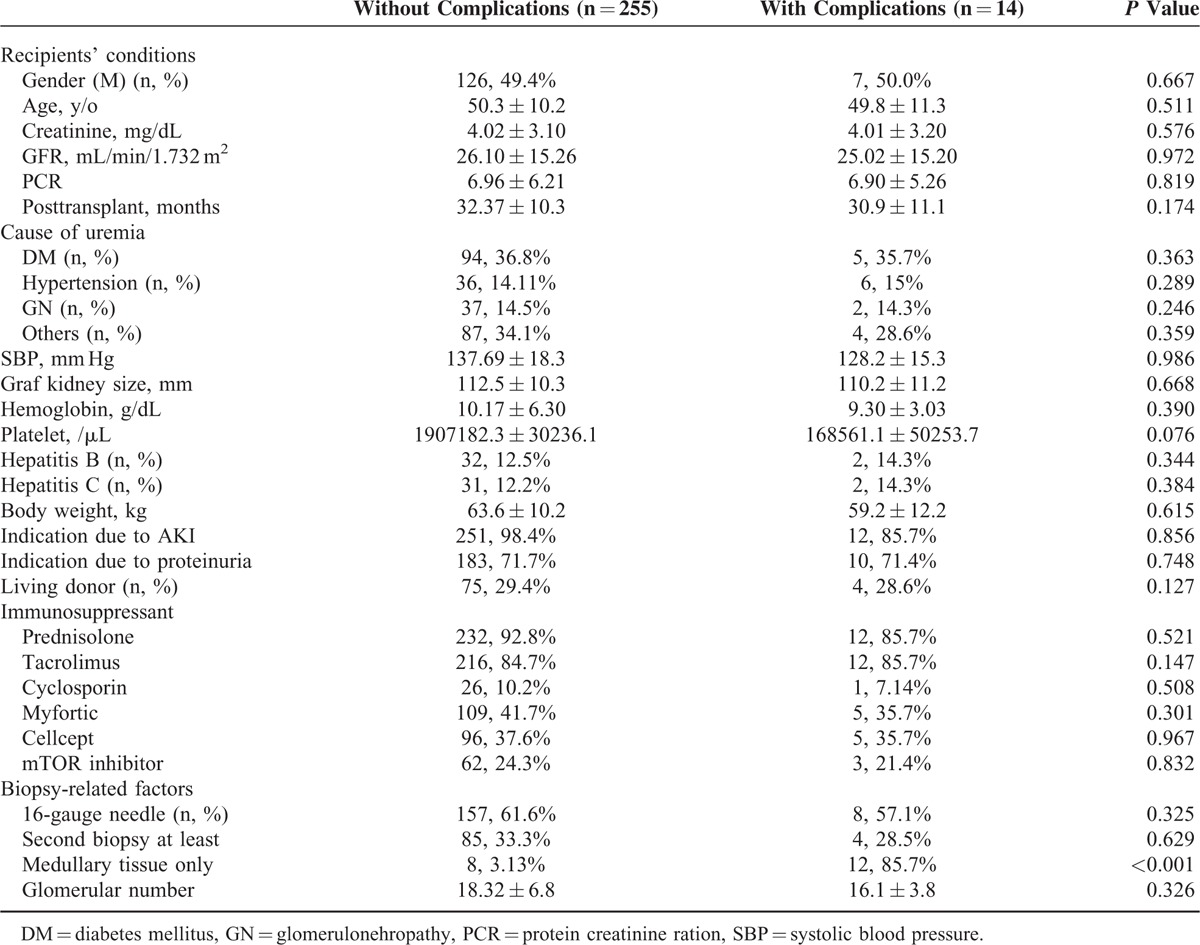
Difference Between Recipients With or Without Complications

## DISCUSSION

Renal biopsy is the final diagnosis for kidney disease, including native and allograft kidneys. It is an essential tool for diagnosis and treatment, especially in cases of renal transplantation. The safety and complications in native kidney biopsy have been well defined.^5^ However, studies on allograft kidney biopsy are still lacking. The largest study on allograft kidney biopsy was conducted in 2003,^9^ which included 2127 cases. The study reported that the incidence of clinically significant complications after protocol biopsy of a stable renal transplant was low. However, the study was conducted than 10 years ago and all of the studied cases were protocol allograft kidney biopsy. Recently, but also already 10 years ago, a study with transplant protocol biopsies was done and there were 508 patients.^13^ Patients receiving protocol biopsy are more stable than patients receiving biopsy with indication. Biopsy with indication was always performed due to renal function deterioration or proteinuria. The association between renal dysfunction and bleeding was observed more than 200 years ago and the causes are believed to be multifactorial, including defects intrinsic to the platelet and abnormal platelet-endothelial interaction.^14^ That is, the complication rate following allograft biopsy with indication can be expected to be greater than that following protocol allograft biopsy. Moreover, most centers performed allograft kidney biopsy with indication, rather than protocol kidney biopsy. Unfortunately, there are relatively few data on the safety and complications in allograft kidney biopsy with indication. Also, the complication of allograft kidney biopsy is time-dependent owing to the need for application of medical devices, including sonography to guide biopsy, automated spring-loaded biopsy device, and usage of Desmopressin. Therefore, there is a need to determine the risks and complications rates of allograft indication kidney biopsy in the modern era. A meticulous review of the recent literature within the past 10 years only described 1 study on the safety of allograft kidney biopsy.^8^ Furthermore, the study only included children and adolescents and did not differentiate between indication and protocol biopsy. Our patient population is the largest indication allograft kidney biopsy cohort in the modern era. Consequently, the results of the present study may be of value to clinicians.

As compared to allograft “protocol” biopsy,^9^ the complications of allograft indication biopsy are as follows: hematoma (1.11% vs 2.6%), gross hematuria (2.23% vs 1.9%), arteriovenous fistula (0 vs 2.4%), graft loss (0% vs 0.04%), blood transfusion (0.74% vs 0.14%), radiological intervention (0.37% vs 0.04%), bowel perforation-related peritonitis (0 vs 0.09%), and surgical intervention (0 vs 0.09%). For the allograft indication biopsy cohort, there was a greater number of patients with gross hematuria, need for blood transfusion, and radiological intervention. Despite the higher incidence of radiological intervention in the indication allograft biopsy group, the number of such cases in both studies was just one. We believe it is very rare to require radiological intervention regardless of the type of biopsy. Much more severe complications, such as graft loss and bowel perforation, were also much lower in this indication study. In summary, we believe that even with a greater need for blood transfusion, allograft biopsy with indication did not have more severe complications than those of allograft protocol biopsy. Unexpectedly, these results showed that the risk of severe complication following a biopsy of a “normal kidney” was higher than that following a biopsy of an “inflamed kidney.” In a study by Furness et al,^9^ 318 patients (15.0%) were only observed for 4 hours after allograft biopsy, which we think is inadequate. In the present study, all patients were kept under observation in the hospital for at least 24 hours and followed up for up to 1 week in the outpatient department. As such, we suspect that the complication rate in the aforementioned [?] study may have been underestimated. Allograft kidney biopsy with indication in our protocol had a very low complication rate. Compared to another study on protocol allograft kidney biopsy,^15^ 7 patients (2.7%) experienced severe complications: gross hematuria with obstructive acute renal failure in 6 cases and isolated gross hematuria in 1 case. The complication rate was also more than our study (2.7% vs 2.23%).

Within the past 15 years, the only study on allograft kidney biopsy with “indication” was conducted by Nicholson et al.^16^ All of the biopsies were performed for acute kidney injury or to monitor the response to antirejection treatment. Size of biopsy needle (14, 16, or 18 gauge) was not a risk factor for complications.^16^ The macroscopic hematuria rate was 8%, which was higher than ours (2.23%) but similar to other previous reports from 15 years ago.^17–22^ Two studies reported the rate of gross hematuria was around 3%,^23,24^ but they were done more than 30 years ago. Therefore, in the current era, our study had the lowest complication rates following allograft kidney biopsy with indication. In the past 30 years, there were 7101 renal biopsies, which is the highest number among medical institutes nationwide. Our hospital also has the 3rd highest number of cases of renal transplantation in the country.

Regardless of the type of biopsy, allograft with “indication” or “protocol” kidney biopsy, our protocol had the lowest complications rates. There are a number of reasons which may explain this excellent outcome. First, allograft kidney biopsy was always performed by experienced nephrologists in our hospital. All biopsies were done with the help of real-time sonography-guidance and automated spring-loaded biopsy device. Second, before allograft kidney biopsy, we normalized blood pressure and coagulation function to the greatest extent possible. Any anticoagulation medication was strictly restricted before and after allograft kidney biopsy. Last, every recipient received 4 vials of Desmopressin 30 minutes before biopsy. This is the 1st study to report the use of regular prophylactic Desmopressin, which is an analog of antidiuretic hormone with little vasopressor activity. It can improve the bleeding time or in vitro tests of platelet dysfunction by increasing the release of large factor VIII:von Willebrand factor multimers from endothelial cells.^25^ To date, there have been no studies on the use of Desmopressin in allograft kidney biopsy, but use of this drug was reported in a study of native kidney biopsy.^26^ It was a single-center randomized placebo-controlled trial in which 162 patients were randomly assigned to either Desmopressin or placebo prior to biopsy. The result revealed Desmopressin can decrease the risk of bleeding and hematoma size even with better renal function: all patients had an eGFR greater than 60 mL/min per 1.73 m^2^, blood pressure less than 140/90 mm Hg, and normal coagulation parameters. However, adverse effects of Desmopressin, such as thrombosis or hyponatremia, were not discussed in that study. Moreover, no studies have mentioned the application of Desmopressin in allograft kidney biopsy. Our study is the 1st to report the routine administration of Desmopressin before allograft biopsy and to date we have had the lowest rate of complications. No thrombosis or hyponatremia was noted in our study.

To the best of our knowledge, no study has shown potential predictors of postbiopsy bleeding complications in allograft kidney biopsy in the current era. In the univariate logistic regression model, under the condition of normal coagulation, no recipient conditions, biopsy report findings, or size of biopsy gun were associated with postbiopsy bleeding. Pathological tissue containing “medulla only” was the only factor predicting postbiopsy bleeding (*P* < 0.001), which is compatible with the result of a study by Beckingham et al.^22^ “Medulla only” suggests no clear identification of the renal capsule. The needle possibly punctured through the entire cortex to the border of the medulla. There are many large branches of vessels and collecting tracts over the inner medulla. The reason that an insufficiently thick renal cortex may be a risk factor in renal biopsy could be due to the potential for medullar injury. Therefore, in order to avoid complication it is vital to identify the renal capsule, differentiate between the renal cortex and medulla by sonography, and then make a very superficial pass of the needle. Once the needle tip has been clearly shown to be located near renal capsule and avoid any medullar injury, other factors will not be associated with postbiopsy bleeding.

There were some limitations in this study. First, the study size was relatively small. However, our study size was the biggest within 20 years for allograft kidney biopsy. Second, to clarify the benefit of Desmopressin, a prospective double-bind study to compare the Desmopressin group and placebo group is needed. Third, we did not routinely collect the serum sodium before and after biopsy. To determine any possible complications of Desmopressin, we recommend that future studies measure serum sodium. However, hyponatremia is seldom reported after administration of 1 dose of Desmopressin. Also, we checked serum sodium if patients complained of nausea or vomiting. Last but not least, we did not record the path-histological results in this article. Some people may wonder that path-histological findings may be associated with the complications of allograft biopsy. However, pathologists read the allograft kidney biopsies based on “Banff classification,” which was modified in 2013.^27,28^ In other words, it is still evolving and changing and there are no absolutely definite criteria for diagnosis. Thus, we did not record the variables of pathological diagnosis in this article.

## CONCLUSION

In the modern era, allograft kidney biopsy with indication is safe with acceptable complication rates. Our meticulous survey of coagulation function, routine use of prophylactic Desmopressin, and sonography guidance to avoid biopsying deep in the medulla or medullar tissue only showed that our allograft indication biopsy protocol had the lowest complication rates compared with previously reported rates.
